# A component method to delineate surgical spine implants for proton Monte Carlo dose calculation

**DOI:** 10.1002/acm2.13800

**Published:** 2022-10-09

**Authors:** Chih‐Wei Chang, Serdar Charyyev, Joseph Harms, Roelf Slopsema, Jonathan Wolf, Daniel Refai, Tim Yoon, Mark W. McDonald, Jeffrey D. Bradley, Shuai Leng, Jun Zhou, Xiaofeng Yang, Liyong Lin

**Affiliations:** ^1^ Department of Radiation Oncology and Winship Cancer Institute Emory University Atlanta Georgia USA; ^2^ Department of Radiation Oncology University of Alabama Birmingham Alabama USA; ^3^ Department of Neurosurgery Emory University Atlanta Georgia USA; ^4^ Department of Orthopaedics Emory University Atlanta Georgia USA; ^5^ Department of Radiology Mayo Clinic Rochester Minnesota USA; ^6^ Department of Biomedical Informatics Emory University Atlanta Georgia USA

**Keywords:** Monte Carlo dose calculation, proton therapy, surgical implant delineation

## Abstract

**Purpose:**

Metallic implants have been correlated to local control failure for spinal sarcoma and chordoma patients due to the uncertainty of implant delineation from computed tomography (CT). Such uncertainty can compromise the proton Monte Carlo dose calculation (MCDC) accuracy. A component method is proposed to determine the dimension and volume of the implants from CT images.

**Methods:**

The proposed component method leverages the knowledge of surgical implants from medical supply vendors to predefine accurate contours for each implant component, including tulips, screw bodies, lockers, and rods. A retrospective patient study was conducted to demonstrate the feasibility of the method. The reference implant materials and samples were collected from patient medical records and vendors, Medtronic and NuVasive. Additional CT images with extensive features, such as extended Hounsfield units and various reconstruction diameters, were used to quantify the uncertainty of implant contours.

**Results:**

For in vivo patient implant estimation, the reference and the component method differences were 0.35, 0.17, and 0.04 cm^3^ for tulips, screw bodies, and rods, respectively. The discrepancies by a conventional threshold method were 5.46, 0.76, and 0.05 cm^3^, respectively. The mischaracterization of implant materials and dimensions can underdose the clinical target volume coverage by 20 cm^3^ for a patient with eight lumbar implants. The tulip dominates the dosimetry uncertainty as it can be made from titanium or cobalt–chromium alloys by different vendors.

**Conclusions:**

A component method was developed and demonstrated using phantom and patient studies with implants. The proposed method provides more accurate implant characterization for proton MCDC and can potentially enhance the treatment quality for proton therapy. The current proof‐of‐concept study is limited to the implant characterization for lumbar spine. Future investigations could be extended to cervical spine and dental implants for head‐and‐neck patients where tight margins are required to spare organs at risk.

## 1 INTRODUCTION

With the development of new surgical techniques, there are millions of annual procedures for vascular, dental, spinal, hip prosthesis, and breast implants in the USA.^1^, ^2^, ^3^, ^4^, ^5^ Roughly 40% of patients receiving radiotherapy to areas around the spine have metallic surgical implants. Separate single‐institution studies conducted at Massachusetts General Hospital (MGH) and the Paul Scherrer Institute (PSI), including 50‐sarcoma patients^6^ and 100‐chordoma patients,^7^ respectively, have confirmed that radiation therapy results in lower tumor control rates in patients with surgical implants. Although these disease groups are generally curable with pencil beam scanning proton therapy (PT) in patients without implants,^6^, ^7^, ^8^ photon‐based radiotherapy has been found too toxic because of concerns of excessive dose to surrounding organs at risk, especially the need to preserve the functionality of nerve roots and long‐term trabecular bone loosening^9^, ^10^ indicated by the MGH phase‐2 study.^7^ University of Florida (UF)^11^ integrated some photon components into PT treatment to mitigate dosimetric uncertainty for implant patients. The photon components included either a 9‐field intensity modulated radiation therapy plan with 6‐MV photons or 3‐field forward planning with 6‐ and 15‐MV photons. UF achieved a better local control rate than that reported by MGH^12^ and PSI.^13^ Unlike the other two institutions, no long‐term toxicities about nerve roots and trabecular loosening were reported in the UF study.

Rutz et al.^14^ suggested that patients with large weight‐bearing implants require more precise imaging methods and proton Monte Carlo dose calculation (MCDC) rather than analytical dose calculation because MC is more accurate for particle transport in heterogeneous tissues.^15^, ^16^, ^17^, ^18^, ^19^ As MC was not widely clinically available in commercial treatment planning systems until recently, NRG Oncology launched a survey^20^ to check the readiness of proton centers to implement MC in clinical trials. Based on the survey, NRG recommended the implementation of MC to better model human tissue heterogeneity for patients without implants. For the implant patients, the consensus workflow is to acquire computed tomography (CT) images with metal artifact reduction (MAR) algorithms and then use material overrides for surgical implants and remaining artifacts in the surrounding tissues so that artifacts do not inhibit dose calculation.

Determination of the true three‐dimensional (3D) geometry and material composition of surgical spine implants is especially crucial for PT because a screw component can be of various shapes and composed of titanium or cobalt–chromium alloys (Chromalloy).^21^, ^22^ Chromalloy is often chosen due to the need for strength in the weight‐bearing function of the lumbar spine. Inadequate characterization of these surgical implants can impact the quality of the PT treatment and potentially the outcome. To alleviate the negative impact of the implants, treatment techniques often include the use of seven fields^8^, ^11^ with treatment plan robustness of 5 mm/5% to cover using range uncertainties up to 10 mm^23^ and scatter uncertainties.^24^ However, these studies assume that the implant details are known, and there is no uncertainty for implant geometry or materials.^20^, ^23^, ^24^

Similarly, such accurate implant characterization was assumed by multiple groups^25^, ^26^, ^27^, ^28^, ^29^ in the generation of sinogram data and used to train various neural networks about metal artifacts. To our best knowledge, current artificial intelligence (AI) publications have reported the success of MAR in digital patients with assumed implant materials and geometry artificially added. As the artifacts vary with implant materials and geometry, a realistic implant characterization method would be vital to AI‐assisted MAR technology to better simulate artifacts for training data to apply from digital phantom to real patients.

The accuracy of MC dose calculation is dominated by material characterization, which is typically carried out using a stoichiometric calibration that fits standard 12‐bit CT Hounsfield units (HUs) to known human tissues.^30^, ^31^ For metal implants, pioneer studies utilizing an extended 16‐bit CT scale showed advantages in representation of HU, in addition to determining composition for hip prosthesis^32^, ^33^ and mitigating CT artifacts to have more accurate conventional photon treatment plans.^34^ As metal implants preferentially absorb the low‐energy photon portion of the bremsstrahlung CT spectrum because of their higher effective atomic number and physical density. The extended 16‐bit HU of these implants is subject to stronger beam hardening than any human bony tissue. Therefore, the extended HU values reported for hip prosthesis^32^ will be different from that of lumbar and cervical spines for the same metal materials due to the sizes of implant and body cross section. Blind application of previously published extended HU could lead to a potential mischaracterization of implant materials and volumes for a new clinical site.

Patients with spine chordomas and chondrosarcomas usually have surgical implants in the clinical target volume (CTV), and the implants can cause potential proton range and scattering uncertainties due to inaccurate implant delineation and unknown implant materials. In this study, we propose a surgical implant component delineation method using partial prior knowledge, extended HU, and fine resolution CT reconstruction to conserve the geometric features of the implants. We intend to report the potential geometric volume and dosimetric improvements of a clinical treatment plan using our proposed procedures over the current clinical practice.

## 2 MATERIALS AND METHODS

**FIGURE 1:**
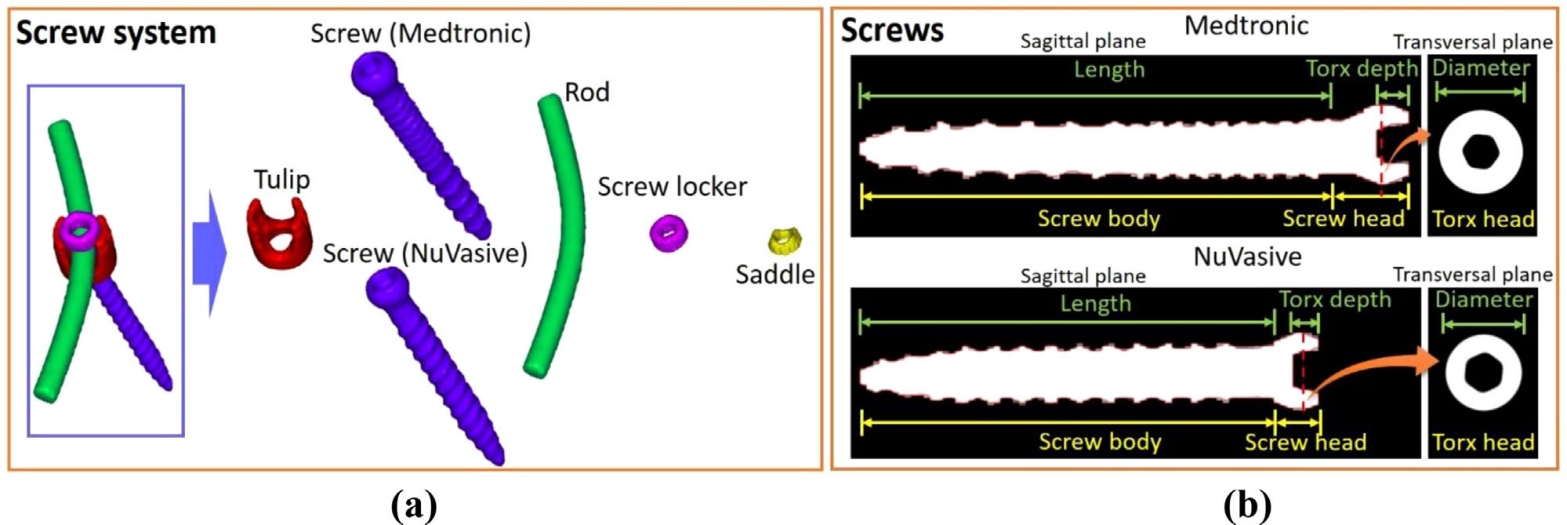
(a) 3D rendered contours of spine screw components from computed tomography (CT) images with RECON 125, and (b) definition of screw structures for different vendors

Some screw components, such as tulips and rods, can be made from either titanium or Chromalloy. As seen in Figure 1, the volumes or dimensions of these components can vary from vendor to vendor. We collected two spine screw systems, Medtronic CD HORIZON SOLERA multiaxial and NuVasive RELINE POLYAXIAL series with model numbers 55840006545 and 13016540. Rod models are 1553201070 and 1435507 for Medtronic and NuVasive, respectively. The Medtronic tulip is made from Chromalloy based on the American Society for Testing and Materials (ASTM) F1537^35^ with the composition of 66% Co, 28.26% Cr, 5.5% Mo, 0.2% Ni, and 0.04% C. All other screw units are made from titanium based on ASTM F67^36^ with the composition of 98.96% Ti, 0.5% Fe, 0.4% O, 0.08% C, 0.05% N, and 0.01% H.

### 2.1 Component method for surgical implant contours

The first step of the component method is to build a library including contours for each component of the spine screw system from CT images with extended HU. This artifact‐free component library, in predefined 2D contours on different planes, was used for direct image registration in phantom and patient images as an alternative to direct implant delineation on artifact‐contaminated CT images. We will report the uncertainty of implant volume due to image registration. The component contours are obtained by taking separate CT scans for each component and then adjusting CT number threshold values on the reconstructed images to match the known volumes of each screw unit. Figure 1a illustrates the 3D rendering of a spine screw system, delineated from CT images using a Siemens Definition Edge scanner with a slice thickness of 0.6 mm and an axial resolution of 0.24 mm from a field‐of‐view (FOV) of 125 mm (RECON 125). The small FOV allows for a fourfold decrease in in‐plane resolution compared to a typical FOV of 500 mm. Figure 1b shows CT sagittal views used to define the length of the screws. Both screws can be further decomposed into the head and body. The screw heads are semispherical and use the Torx^26^ design, which includes a six‐point star‐shaped pattern with the size of T25, that is, a maximum point‐to‐point distance of 4.5 mm. The fine resolution of RECON 125 CT images allows contours to capture the curvature to accurately reflect the shapes of implant components. A precision scale was used to gauge the weight of each component and determine the volume using vendor‐provided mass densities. These component weights were later used to derive reference volumes to determine the threshold of extended HU to get the correct image rendered volume and report the minimum, mean, and maximum HU in each study scenario. Typically, there are very limited choices of implant components per vendor. Artifact‐free standalone CT scans of the tulips can clear potential confusions of implant material and volume that tend to arise due to the combination of tulip, rod, locker, saddle, and screw head.

**FIGURE 2:**
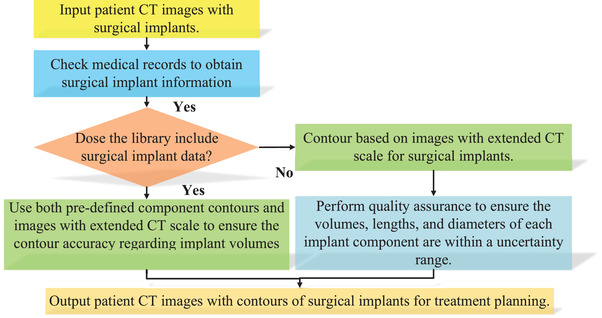
Workflow of a component method to delineate surgical implants from images with extended computed tomography (CT) scale

Figure 2 depicts the workflow of the component method for surgical implant contours. Once the patient images are acquired, medical records are checked to obtain surgical implant details, such as implant model numbers, materials, and essential dimensions like screw lengths and rod diameters. Then we can search the component contour library for patient‐specific surgical implants. If the specific implant exists in the library, we can use both predefined component contours and images with an extended CT scale to ensure contour accuracy regarding implant volumes and shapes on patient images. If the library does not contain the specific implant, it can be delineated by using an HU threshold and ensuring implant contours conserve the dimensions of key features, such as tulip volumes, screw lengths, and rod volumes, within a satisfactory uncertainty range. Ultimately, patient images with contours and implant material overrides are used for treatment planning.

### 2.2 Quality assurance for contour rationality using partial prior knowledge

The medical record usually provides some partial prior knowledge about the surgical implant information, such as lengths of spine screws and diameters of screws and rods. Such information can be converted into volumes for screw bodies and rods, which can be used for quality assurance. An accurate implant volume estimation can reduce the uncertainty for proton dose calculation, mainly when the CTV includes surgical implants. For patient studies, screw heads given in Figure 1b are embedded in tulips, and it is difficult to segment the two components separately from CT images. Therefore, we check the volume of screw bodies given in Figure 1b to ensure the accuracy of implant contours. Although spine screws are positioned in 3D space, we can rotate the screw contours to make its central axis perpendicular to the transversal CT plane for screw length measurement. The following equation defines the relative uncertainty of screw length where *L*, *c*, and *ref* are the length, contour, and reference, respectively:

(1) $$\mathrm{Inaccuracy}\;\mathrm{of}\;\mathrm{screw}\;\mathrm{length}\;=\;\left|L_{c}-L_{ref}\right|$$

**FIGURE 3:**
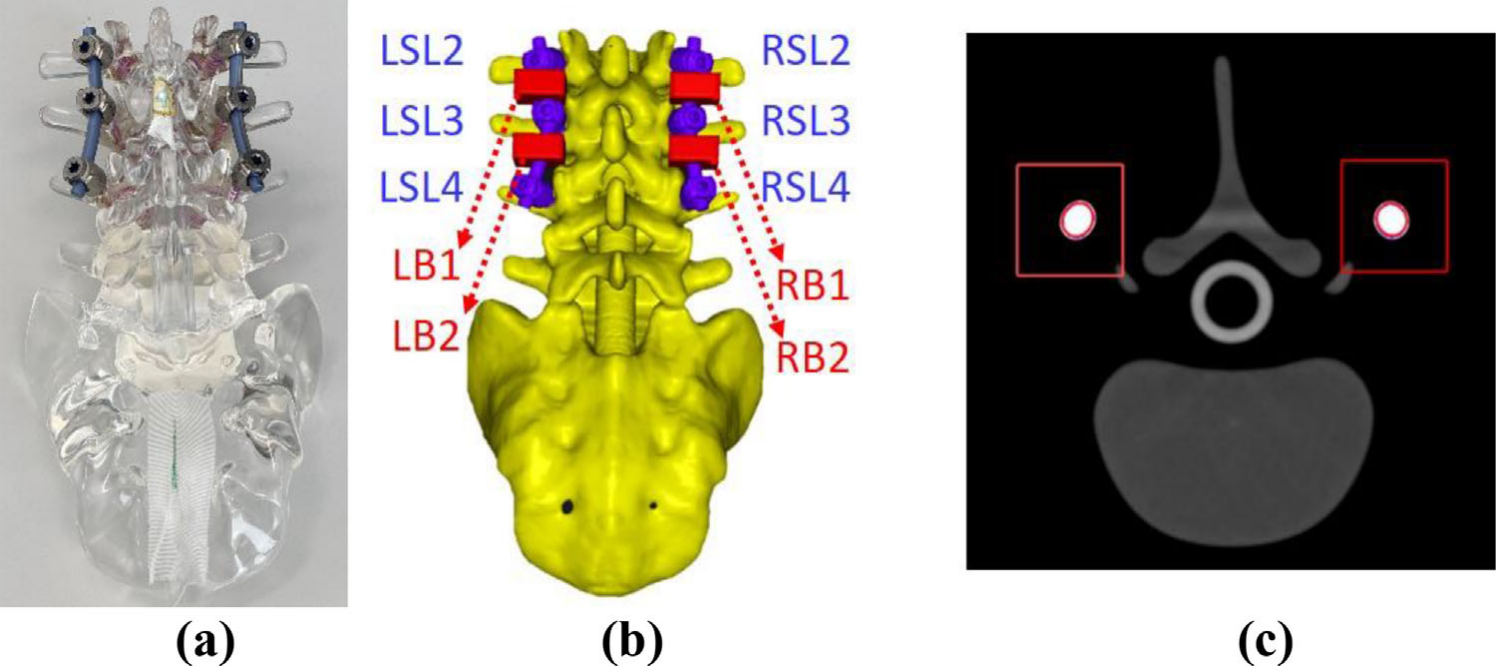
(a) A photograph of the spine phantom for surgeon training; (b) 3D contours of the spine phantom with NuVasive surgical screw system, rendered from (c) computed tomography (CT) images with RECON 125

Rods are bent along to follow the spinal curvature, which makes a direct measurement of rod diameters difficult. Therefore, we use a set of predefined 1‐cm long bounding boxes with the cross section of 2 × 2 cm^2^ to perform quality assurance for contour rationality of rods as given in Figure 3. Within these left and right bounding boxes (LB and RB), we can define cylindrical reference contours with actual implant rod diameters using prior knowledge, and we can register those reference contours to implant rods within LB and RB. The rod volume uncertainty can be measured by the following equation where *V_box_*, *V_rod_*, and *V_ref_* are volumes of bounding boxes, implant rod contours, and cylindrical reference contours, respectively:

(2) $$\mathrm{Inaccuracy}\;\mathrm{of}\;\mathrm{rod}\;\mathrm{volume}\;=\left|\left(V_{box}\cap V_{rod}\right)-\left(V_{box}\cap V_{ref}\right)\right|\;$$

### 2.3 Application of extended HU method in standalone implant component and patient

Our standard clinical practice is based on using a threshold value of 2400 HUs to delineate surgical implants from the standard 12‐bit CT images, and the method is recommended by literature.^23^ According to a recent NRG survey,^20^ this practice can vary from clinic to clinic. In this study, we have found that we could not report a universal threshold for each metal material. Instead, minimum, mean, and maximum HUs are reported for each scenario of titanium and Chromalloy, in agreement with the methods reported in the literature.^32^, ^34^

### 2.4 Evaluation of dosimetric consequences from inaccurate implant materials and volume

A patient with the Medtronic screw system installed in the lumbar spine (L1, L2, L4, and L5), including eight tulips, eight screws, and two rods, was treated for chordoma. In addition to the original treatment plan, two dose calculations were performed: the first to evaluate the dosimetric impact of inaccuracies in the overall implant characterization, that is, the characterization of both component material and volume, and the second of the geometric volume only. In the first scenario, mischaracterization of Chromalloy with titanium was considered. In the second scenario, the material assignment was accurate, and only mischaracterization of the component volume was investigated. All dose calculations were done with the RayStation 9B MCDC algorithm^37^ in using a 1 mm dose grid instead of the conventional 2‐mm dose grid.

## 3 RESULTS

### 3.1 Screw component contours

Table 1 summarizes the measured weights and subsequent volumes of each screw component given in Figure 1a. Each screw component is measured by a scale with a precision increment of 0.01 g, and the volumes can be calculated from densities of titanium and Chromalloy, which are 4.51 and 8.28 g/cm^3^. The CT threshold values are selected to ensure that the component contour volumes are identical to the measurement. Tulips from both vendors have similar volumes of ∼0.8 cm^3^, whereas the volume of screws and rods depends on their length. The rod length is 70 mm for both vendors. Table 2 gives the screw parameters in Figure 1b.

**TABLE 1:** Summary of the volume measurement for screw components and the required computed tomography (CT) threshold values for each contour

	NuVasive					Medtronic				
			Contour HU					Contour HU		
	Mass (g)	Volume (cm^3^)	Min.	Max.	Mean	Mass (g)	Volume (cm^3^)	Min.	Max.	Mean
Tulip	3.52	0.78	4400	10 540	7602	6.76	0.82	10 200	24 850	16 567
Screw	3.49	0.77	4700	12 510	8980	4.47	0.99	4700	12 540	9017
Rod	7.49	1.66	4800	11 140	9382	8.03	1.78	4800	12 200	9710
Screw locker	0.93	0.21	4400	11 510	7869	0.85	0.19	4500	10 720	7737
Saddle	0.33	0.07	4200	9610	6444	0.37	0.08	4000	11 070	6539

Abbreviation: HU, Hounsfield unit.

**TABLE 2:** Summary of the screw parameters listed in Figure 1

	Screw length (mm)	Torx depth (mm)	Torx diameter (mm)	Screw body volume (cm^3^)
NuVasive	40	2.59	7.24	0.66
Medtronic	45	3.46	7.51	0.81

### 3.2 Component method for surgical implant delineation from a spine phantom

**FIGURE 4:**
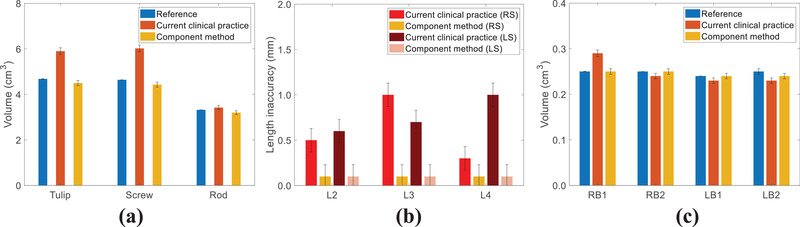
For the spine phantom, comparisons of (a) volumes for six tulips, six screws, and two rods; (b) relative length uncertainty for six screws at different spine vertebrae; and (c) rod volumes at four bounding boxes between contours by the current clinical practice and component method

To compare the contour difference between the current clinical practice and the component method, a spine phantom containing the NuVasive screw system was scanned (Figure 3a). For this system, we have predefined contours for each component, as is determined in Section 3.1. Figure 4a shows that the current clinical practice overestimates the combined volume of the six tulips, the six screws, and the two rods by 1.22, 1.38, and 0.10 cm^3^, respectively. The component method underestimates the volumes of these same component groups by 0.18, 0.21, and 0.12 cm^3^. The uncertainty of the reference implant volumes is estimated to be 0.2% by the weight measurement of each screw component, whereas that due to image registration of implant components (tulip, screw, and rods) is 2.5%. Figure 4b shows the relative length uncertainty of three left screws and three right screws at different spine vertebrae ranging from L2 to L4. The current clinical practice can result in a maximum inaccuracy of 1.0 mm for screw lengths. Figure 4c presents the comparison of rod volumes in each bounding box given in Figure 3b. The current clinical practice and component method show a maximum discrepancy of 0.04 and 0.01 cm^3^ from the reference.

### 3.3 Component method for a patient plan

**FIGURE 5:**
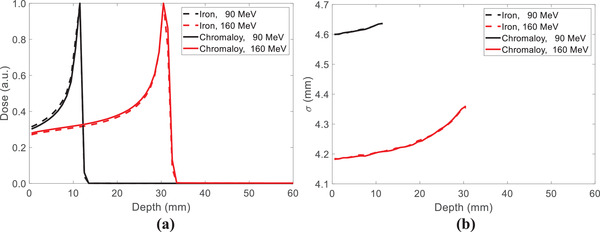
Comparisons of (a) proton ranges and (b) spot profiles in 40 × 40 × 40 cm^3^ iron and Chromalloy volumes using TOPAS with 90 and 160 MeV proton spots

As Chromalloy is not available in RayStation 9B, iron with a density of 8.17 g/cm^3^ is used in RayStation. Figure 5 demonstrates that iron has similar material characteristics as Chromalloy regarding proton ranges and scattering simulated by TOPAS with 90 and 160 MeV proton spots.

**FIGURE 6:**
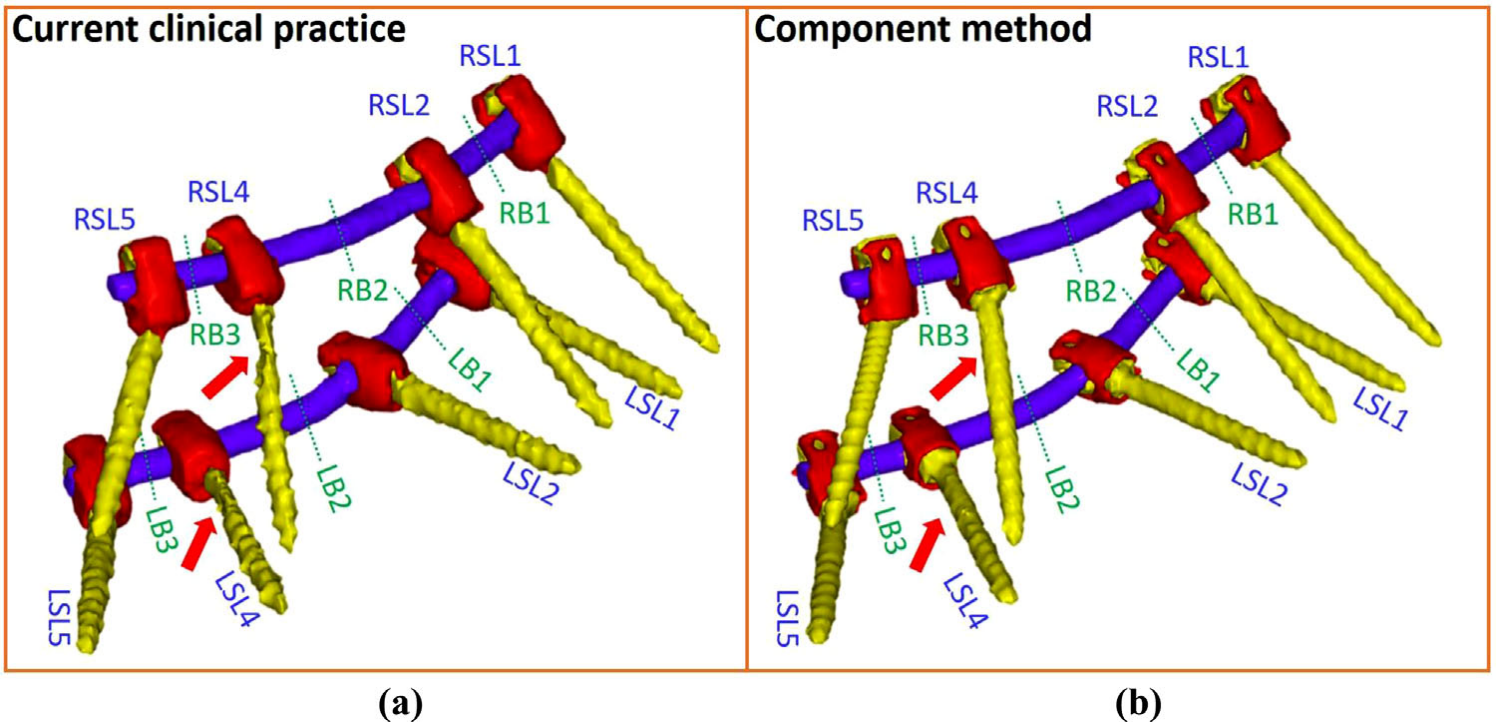
3D rendered surgical implant contours by (a) the current clinical practice and (b) component method for a patient plan

Figure 6 shows the 3D rendered surgical implant contours by the current clinical practice and the component method. The clinical contours involve volume defects, as indicated by the red arrows in Figure 6a. The red tulip contours in Figure 6b are registered from predefined structures from the component library. Overestimation of tulip and underestimation of screw volumes can be observed in current clinical practice.

**FIGURE 7:**
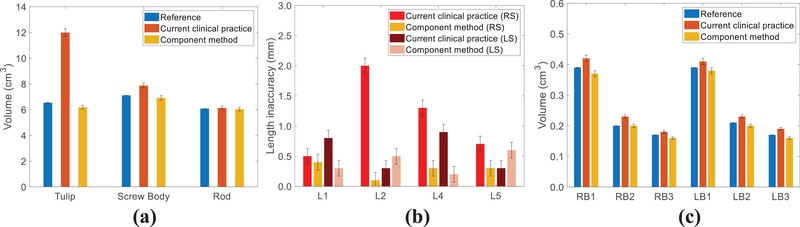
For the patient, comparisons of (a) volumes for eight tulips, eight screw bodies, and two rods; (b) relative length uncertainty of eight screws at different spine vertebrae; and (c) rod volumes at six bounding boxes between contours by the current clinical practice and component method

Figure 7a depicts that the current clinical practice overestimates the combined volumes of eight tulips, eight screw bodies, and two rods by 5.46, 0.76, and 0.05 cm^3^, respectively, whereas the component method underestimates those by 0.35, 0.17, and 0.04 cm^3^, respectively. Figure 7b shows the maximum screw length uncertainties are 4% and 1.2% for the current clinical practice and component method. Figure 7c depicts the comparison of rod volumes in bounding boxes (LB and RB) located at the six locations given in Figure 6. The current clinical practice overestimates the rod volumes by an average of 0.02 cm^3^, whereas the component method underestimates the rod volumes by an average of 0.01 cm^3^. The uncertainties of this patient were estimated to be similar to that of the spine phantom.

**FIGURE 8:**
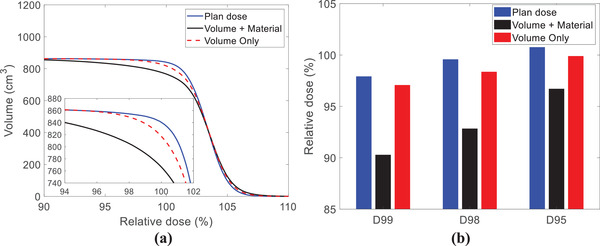
(a) DVH comparisons of surgical implant contours and (b) dose statistics of clinical target volume (CTV) by the current clinical practice with titanium tulips (blue solid lines and bars), current clinical practice with Chromalloy tulips (black solid lines and bars), and component method with Chromalloy tulips (red dashed lines and bars)

**FIGURE 9:**
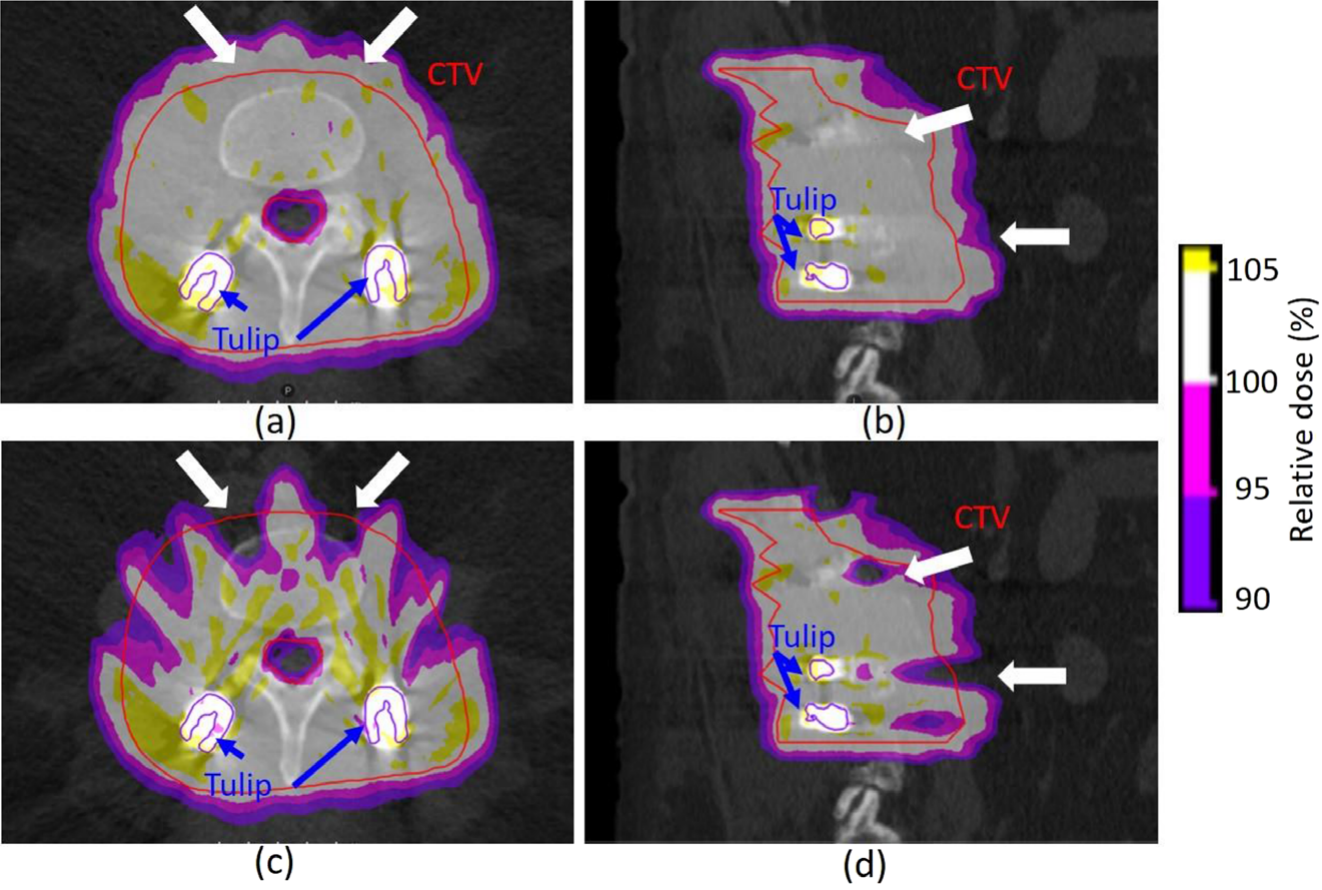
Dose coverage maps of the intended clinical case in (a) transversal and (b) sagittal planes. Dose coverage maps of the hypothetical case in (c) transversal and (d) sagittal planes

For the implant with eight screws and tulips, the cumulative implant volume is about 20 cm^3^. The uncertainty of the implant volume estimation might have dosimetric consequences. Figure 8a depicts the dose–volume histograms for the two additional dose calculations using different implant materials and volume estimation as described in Section 2.4. Figure 8b shows the dose coverage of the CTV in the different scenarios. The volume + material case (hypothetical case using current clinical practice with Chromalloy tulips) shows that *D*_94%_ corresponds to the CTV volume of 840 cm^3^, whereas the *D*_94%_ of the nominal plan corresponds to the CTV volume of 860 cm^3^. The volume + material case includes at least 20 cm^3^ (∼2% of the CTV), which is not covered by 95% of the prescription dose. Such underdose is much smaller for the volume case (component method with Chromalloy tulips). Figure 9 illustrates that the volume + material case could impact treatment margin, resulting in less dose to the CTV than intended, somewhat similar to other reported systemic proton range uncertainties.

## 4 DISCUSSION

The inclusion of tulip contours in the library can reduce the uncertainty of treatment planning for patients with spine screws because screws with the same function but different lengths can share the identical tulip for the same vendor. CT images with high resolution are helpful in determining curvatures of component contours for surgical implant screws that can minimize implant shape uncertainty. The introduction of high‐resolution reconstruction using a 125 mm FOV can characterize implant components at 0.24 mm in plane resolution and 0.6 mm slice thickness. Screw length can also be verified with fine‐resolution CT images as the tapered threads of screws can be seen. However, the super‐resolution images are compromised in patients due to limited kV/mAs and patient scatter.^23^, ^24^, ^31^, ^34^, ^38^ Furthermore, the 0.24‐mm super‐resolution CT image sets are 16 times the size of a conventional image set. Adaptive CT image resolution would be encouraged for TPS vendors to allow more accurate modeling of implant materials and volume. The 1‐mm dose grid is found to generate similar dose distributions to that of the conventional 2‐mm dose grid, most likely due to the size of proton spots.^17^

Determination of the tulip material used for spinal implants is important because various high‐density materials, which ultimately impact the dose calculation, can be used. These tulips can be made from either titanium or Chromalloy, and the total volume of tulips depends on the number of screws installed in patients. Figure 8b shows that CTV *D*_95%_ of the volume + material case is ∼3.3% lower than the prescription dose. According to our institutional standard, replanning may be required as the variation between CTV *D*_95%_ and the prescription dose exceeds 3%. Future investigation will likely investigate robust independent multifield optimization using MCDC^39^ and beam‐specific target volume^40^ to avoid directly shooting surgical implants. Collecting experimental data is also essential to accurately quantify the dosimetry impact of metallic implants by comparisons with independent noncommercial MCDC^41^ to allow more flexible implant characterization and experiment.

**FIGURE 10:**
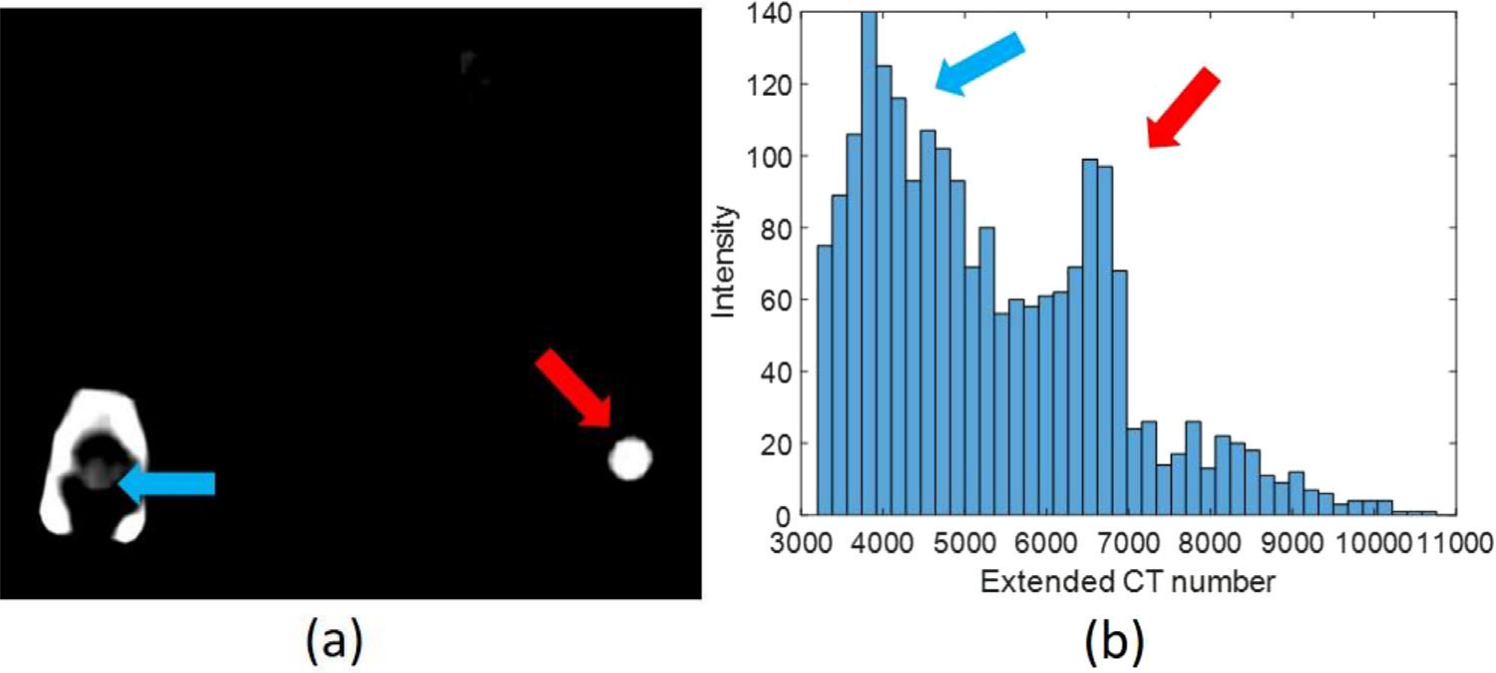
(a) A patient computed tomography (CT) image with extended CT scale, a 125‐mm‐reconstruction diameter, and adjusted window level to emphasize the implant position; (b) the histogram of extended CT scale for all spine screw system in a patient

The current clinical practice tends to overestimate tulip volume (Figure 6) due to the threshold selection of 2400 HU for metal implant contours. We have found that such a fixed threshold in the standard CT scale is not reliable; instead, a flexible threshold in extended‐CT‐scale images combined with prior knowledge should be used. Without prior knowledge, an extended CT scale can mislead the material determination under some circumstances. Figure 10a shows a patient CT image with an adjusted window level to emphasize surgical implants, and Figure 10b gives the HU distribution of total surgical implants with two peaks around 4000 and 7000 HU. The HU distribution of Chromalloy tulips is close to the 7000 HU peak. However, the HU of titanium rods can contribute to both peaks when rods are in different locations. When a titanium rod is embedded inside a tulip, as indicated by a blue arrow in Figure 10a, the HU of the rod is low, and its HU distribution is close to the 4000 HU peak in Figure 10b. When the rod is outside a tulip, its HU is high, and its HU distribution is close to the average Chromalloy HU, potentially leading to errors. The absolute value of HU depends on the patient size, implant dimension, and implant position. Table 3 indicates that the rod of the spine phantom and the tulip of the patient have similar mean HU values due to the difference in patient sizes. The mean HU of titanium screw body at the patient's lumbar of the patient is 4346, whereas the mean HU value of titanium hip prostheses is 7340.^32^ Therefore, the absolute HU should be patient‐specific and site‐specific. Within the same image set, the relative value of extended CT scales potentially can be used to distinguish implant materials if prior knowledge of implant types from vendors is available. The performance of the current method is impacted by the uncertainty due to manual image registration. Future improvement of contour registration can be achieved by deep learning–based image registration methods.^42^, ^43^

**TABLE 3:** Hounsfield unit (HU) statistics within contours of tulips, screws, and rods for the spine phantom and patient

	Contour HU (spine phantom)				Contour HU (patient)		
	Min.	Max.	Mean		Min.	Max.	Mean
Tulip (Ti)	−1120	9600	6485	Tulip (Chromalloy)	470	16 160	7729
Screw (Ti)	−330	10 890	5322	Screw body (Ti)	890	6890	4346
Rod (Ti)	−1090	10 710	7574	Rod (Ti)	1530	8270	5230

A library of surgical implant component contours from various vendors can be built with artifact‐free standalone CT scans, reconstructed with fine resolution, and predefined in TPS to minimize implant contour uncertainty. Such a library can enable component contour registration and conserves the geometrical characteristics and materials of surgical implants. When the prior knowledge of implant contours is not available, the quality assurance of screw lengths and rod volumes is still achievable based on surgical notes. Although screw lengths can vary, the design of tulips is usually generic. We can still verify the order of magnitude for tulip volume, which should be ∼0.8 cm^3^ for a single tulip.

To mitigate metal artifacts by surgical implants, carbon‐fiber‐reinforced polyetheretherketone (CFR‐PEEK) has been implemented in spinal surgery and can achieve similar strength to titanium^37^ with fewer metal artifacts on CT images than titanium implants, leading to a better dosimetric agreement between dose calculation and measurement. However, the replacement of the Chromalloy lumbar spine implant with CFR‐PEEK remains a question due to the need for weight‐bearing function. Furthermore, AI‐assisted MAR in CT can be complemented by MRI.^21^, ^33^ Although MR images have no streaking or flaming artifacts^44^ from the spine implant as in CT, they induce magnetic distortion around the implant, and therefore synthetic CT based on MRI^45^, ^46^ might not replace CT‐based MAR in the near future.

The current study is limited to spine implants in order to determine an optimal workflow. In future studies, we will extend this framework to head‐and‐neck patients, most of whom have dental implants. Future investigations will use the component method to delineate surgical implants as the prior knowledge to regulate deep learning–based MAR models^25^, ^26^, ^27^, ^28^, ^29^ to remedy the material properties of implant surrounding tissues impacted by metal artifacts. Furthermore, dental implants exist in most head‐and‐neck patients and force us to override both implants and surrounding tissues, which requires a tight treatment margin due to the proximity of critical organs. As indicated by the HU of small spine phantom and the lumbar spine patient in Table 3, the mean HU of cervical spine implant and dental implant of head–neck patients are likely to be higher than that reported for the lumbar spine patient. The robustness optimization values need to be adjusted higher than that reported for head–neck patients without implant.^47^

## 5 CONCLUSIONS

By combining prior implant knowledge, extended HU, and a fine resolution reconstruction, a novel component method for surgical implant delineation, was developed for the recently introduced MC in commercial treatment planning systems. It was applied on the screw systems from two major vendors in a spine surgeon phantom and a patient, respectively. The method shows accurate implant characterization, potentially improving proton MC dose calculation for patients with metallic implants.

## AUTHOR CONTRIBUTIONS

Chih‐Wei Chang: method development, manuscript writing, data collection, and processing

Serdar Charyyev: data collection and processing

Joseph Harms: manuscript revising and data collection

Roelf Slopsema: manuscript revising and data collection

Jonathan Wolf: treatment planning

Daniel Refai: phantom design for implants

Tim Yoon: phantom design with implants and advice for surgical implant installation

Mark W. McDonald: physician advice for treatment planning

Jeffrey D. Bradley: physician advice for treatment planning

Shuai Leng: advice for CT scanning and CT imaging reconstruction

Jun Zhou: patient data collection and treatment planning for patients

Xiaofeng Yang: advice for imaging processing and CT denoising

Liyong Lin: supervising the project and research direction and manuscript writing

## CONFLICT OF INTEREST

All authors declare that they have no conflicts of interest.
